# Urea cycle disorder presenting as bilateral mesial temporal sclerosis – an unusual cause of seizures: a case report and review of the literature

**DOI:** 10.1186/s13256-018-1750-8

**Published:** 2018-07-15

**Authors:** Furene Sijia Wang, Denise Li Meng Goh, Hian Tat Ong

**Affiliations:** 1 Khoo Teck Puat - National University Children’s Medical Institute, National University Hospital, Singapore, Singapore; 2 Department of Paediatrics, Yong Loo Lin School of Medicine, National University of Singapore, Singapore, Singapore

**Keywords:** Urea cycle disorder, Mesial temporal sclerosis, Hyperammonemia, Magnetic resonance imaging

## Abstract

**Background:**

Urea cycle disorders are secondary to defects in the system converting ammonia into urea, causing accumulation of ammonia and other byproducts which are neurotoxic. Ornithine transcarbamylase deficiency is the most common of the urea cycle disorders and frequently presents with coma or seizures during hyperammonemia. However, seizures can also occur without metabolic decompensation.

**Case presentation:**

We describe a 23-year-old Chinese woman with urea cycle disorder who presented with confusion due to focal seizures arising from the left frontotemporal region. Interestingly, her ammonia levels remained normal during the seizures. Neuroimaging showed bilateral mesial temporal sclerosis. Her seizures were successfully controlled with two anti-epileptic medications.

**Conclusions:**

This case adds evidence of the predisposition of the temporal lobe to injury in urea cycle disorder. Urea cycle disorder can lead to mesial temporal sclerosis which leads to increased susceptibility of patients to seizures regardless of their metabolic state.

## Background

Urea cycle disorders (UCD) are a group of inborn errors of metabolism caused by the dysfunction of any of the six enzymes or two transport proteins involved in urea biosynthesis. The six enzymes are carbamoyl phosphate synthetase I, N-acetylglutamate synthetase, ornithine transcarbamylase, argininosuccinic acid synthetase, argininosuccinate lyase, and arginase. The two amino acid transport defects are ornithine translocase and aspartate/glutamate carrier. All the UCDs are inherited as autosomal recessive disorders, except for ornithine transcarbamylase (OTC) deficiency. A defect in urea synthesis results in hyperammonemia, which disrupts the aquaporin system and increases astrocyte glutamine synthesis causing brain edema and raised intracranial pressure [1]. The severity of the UCD is influenced by the position of the defective protein in the pathway and the severity of the defect. The clinical presentation is variable with the most severe forms presenting in the neonatal period with seizures, coma, and multiorgan failure. The seizures can be subclinical during acute hyperammonemic episodes. Milder forms of UCD can present at any age with metabolic decompensation or more subtle symptoms such as developmental delay, and behavioral or psychiatric symptoms. However, with good metabolic control of the underlying disease, resultant epilepsy is rare in the course of the disorder [2].

We describe a patient with UCD who presented with seizures despite having good control of her ammonia levels.

## Case presentation

Our patient is a 23-year-old Chinese woman with UCD who presented with seizures 2 years after the latest episode of metabolic decompensation. She was the second child of a non-consanguineous union. Her elder sister and parents were well and there was no history of early deaths in the family, especially male family members. She was delivered at full term via an emergency caesarean section for failure to progress and breech position. Her Apgar was 7 at 1 minute and 8 at 5 minutes, probably due to prolonged maternal anesthesia.

She presented at 14 months of age with gross motor delay and intermittent vomiting after meals. She was alert and interactive. However, she was ataxic and her lower limbs were hypotonic with decreased power and brisk reflexes. The tone, power, and reflexes were normal in her upper limbs. She had intention tremors of the upper limbs. Computed tomography (CT) of her brain did not show any intracranial abnormalities. Her plasma ammonia level was markedly elevated at 327 umol/L (normal range 16 to 53 umol/L). She was treated with intravenously administered sodium benzoate with improvement in the hyperammonemia. She was diagnosed as having OTC deficiency in view of hyperammonemia, elevated glutamine at 1237 umol/L (normal range 400 to 700 umol/L), and elevated urinary orotic acid at 110 mmol/mol creatinine (normal range 0.5 to 3.3 mmol/mol creatinine). Her citrulline level was normal at 17 umol/L (normal range 5 to 60 umol/L). Sequencing of the *OTC* gene did not detect any pathological variant. The inability to identify a pathological variant by sequencing is not unusual. Pathological point mutation variants are found in approximately 80% of patients with enzymatically confirmed OTC deficiency. The remaining patients either have variants in the regulatory regions, variants within the introns, or have large deletions, all of which would not be detected by the sequencing that was done in this patient.

**Fig. 1:**
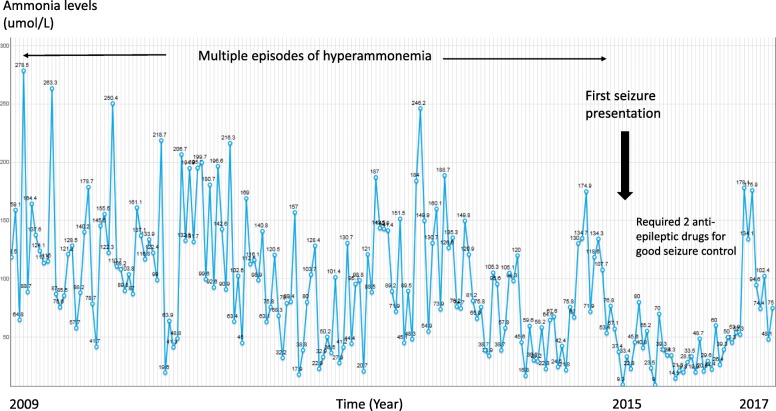
Timeline of serum ammonia levels and clinical progress. Normal range of ammonia being 16 to 53 umol/L

Our patient had multiple hospital admissions from diagnosis to 19 years of age, due to episodes of metabolic decompensation with plasma ammonia levels ranging between 157 and 278 umol/L (Fig. 1). These episodes occasionally occurred due to suboptimal compliance to protein-restricted diet, but most of the episodes occurred without any obvious trigger. She responded each time to intravenously administered sodium benzoate or sodium phenylbutyrate, L-arginine and 10% dextrose infusion with normalization of the ammonia levels. She would then resume her protein-restricted diet, with orally administered sodium benzoate and citrulline. Functionally, she was independent in the activities of daily living. However, she was intellectually impaired with an IQ score of 40 and received special education.

She presented with the first episode of seizure at 21 years of age. She did not have previous febrile seizures in childhood. There was no family history of epilepsy. She had altered mental state and incoherent speech on presentation. The plasma ammonia levels remained normal, ranging from 16 to 45 umol/L.

**Fig. 2:**
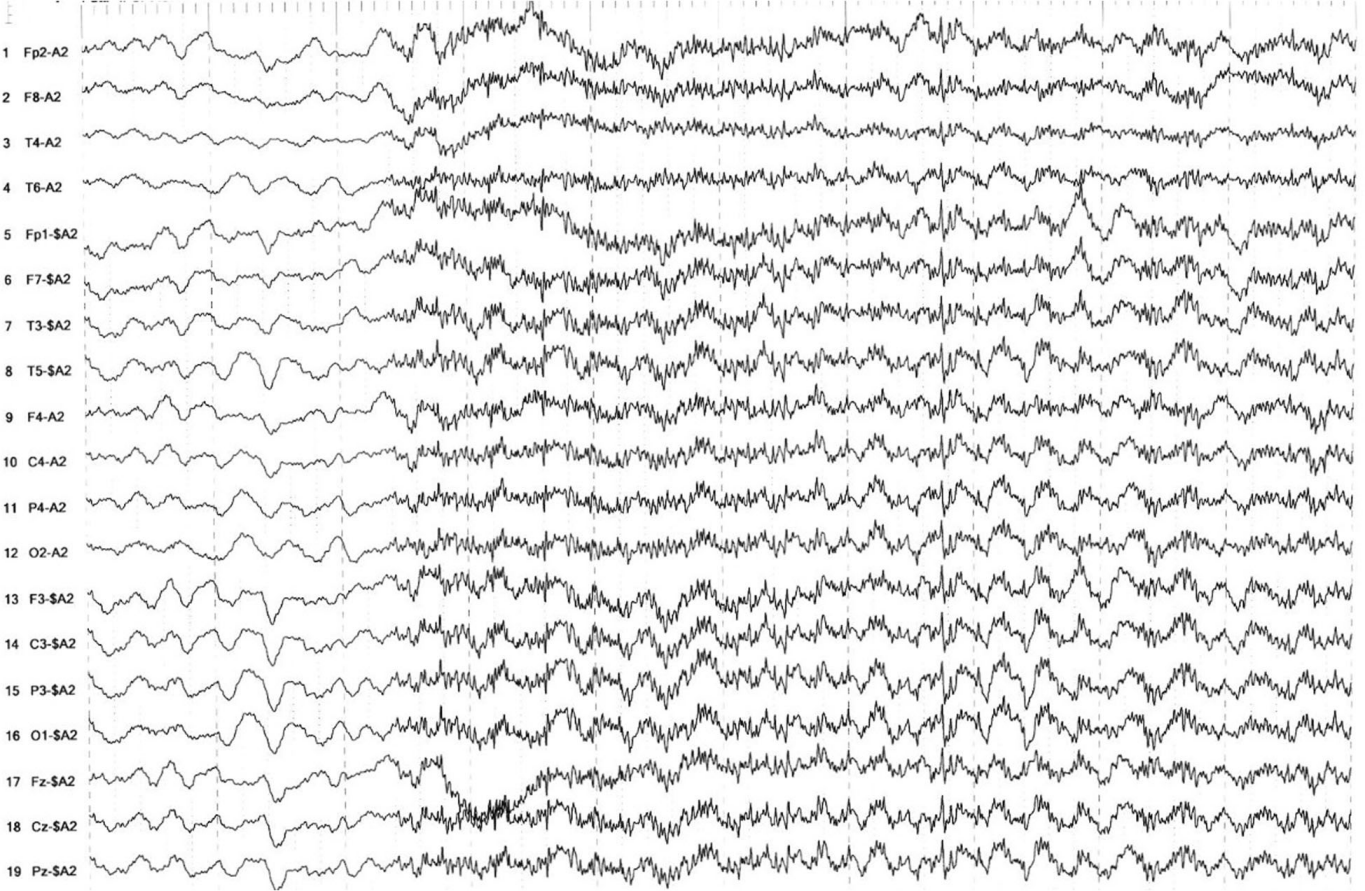
Onset of seizure with runs of generalized rhythmic fast activity

**Fig. 3:**
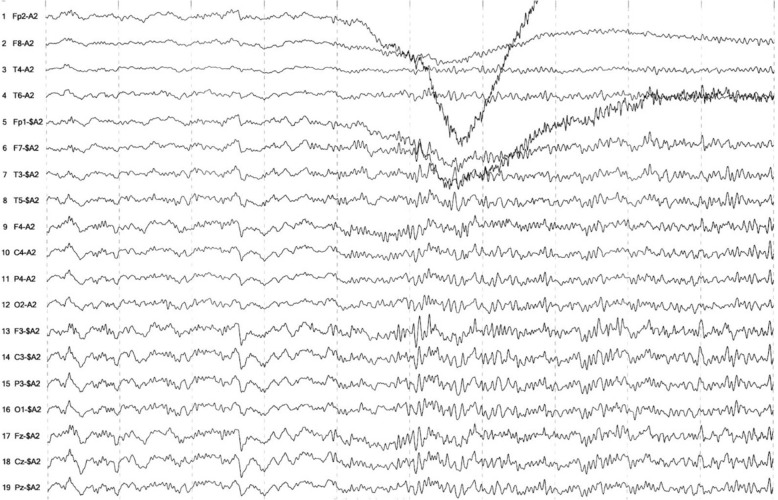
Onset of seizure from left frontotemporal (F3 > T3) region with subsequent generalization

An electroencephalogram (EEG) recorded non-convulsive seizures with the onset of rhythmic fast activity (Fig. 2), occasionally starting at the left frontotemporal region before becoming generalized (Fig. 3). These were associated with clinical manifestations of oral automatisms, impaired consciousness, and right-sided head turn.

**Fig. 4:**
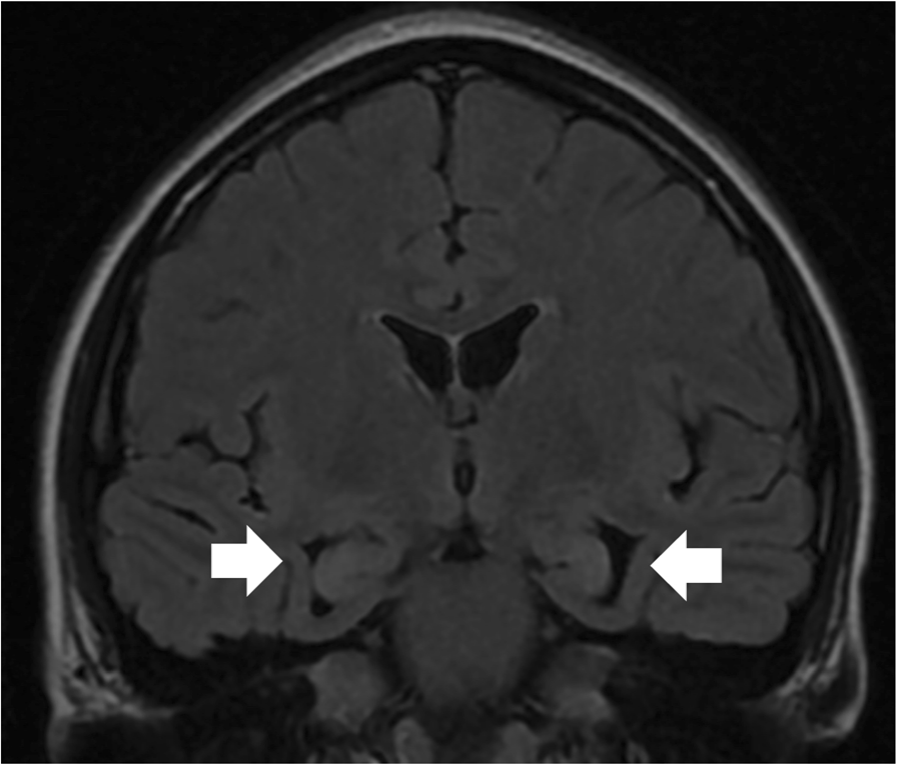
Coronal T2 fluid-attenuated inversion recovery showing signal hyperintensity in bilateral parahippocampal gyri (arrowed) with loss of gray-white matter differentiation due to mesial temporal sclerosis

**Fig. 5:**
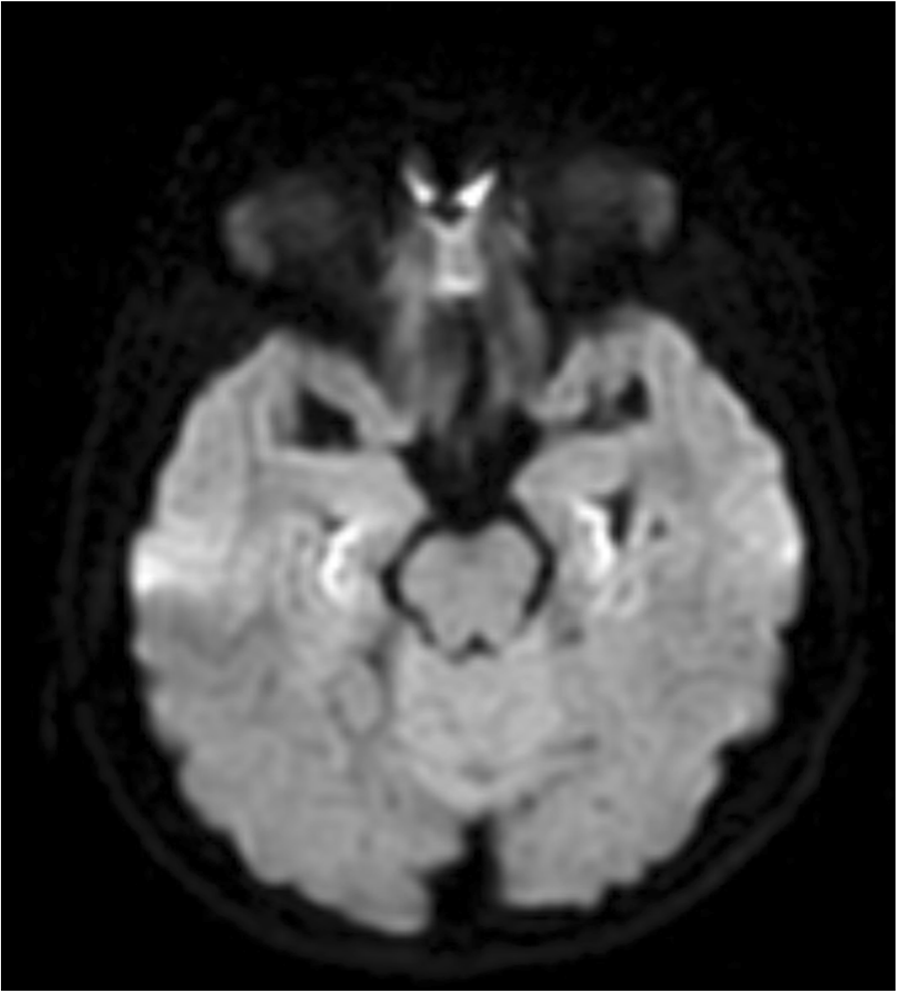
Restricted diffusion in parahippocampal regions on both sides seen on diffusion weighted image sequences

Magnetic resonance imaging (MRI), which was performed 2 days after the first seizure presentation, showed T2/fluid-attenuated inversion recovery (FLAIR) signal hyperintensity in bilateral parahippocampal gyri with loss of gray-white matter differentiation and dilatation of bilateral temporal horns suggestive of hippocampal atrophy, due to mesial temporal sclerosis (Fig. 4). There was also restricted diffusion noted in the parahippocampal regions on both sides (Fig. 5). There was no prior MRI imaging performed. She was started on levetiracetam and pregabalin as these anti-epileptic drugs had minimal drug–drug interaction. Pregabalin was added on as she continued to have breakthrough seizures with levetiracetam. The use of sodium valproate is contraindicated in UCDs as it predisposes to hyperammonemia. Her seizures remained well controlled with the use of the two anti-epileptic medications.

## Discussion

Our patient’s neuroimaging finding of bilateral mesial temporal sclerosis was unusual as acquired mesial temporal sclerosis is commonly known to be a consequence of long-standing refractory epilepsy rather than the cause of seizures. She remained seizure free during multiple episodes of hyperammonemia. However, when the clinical seizures occurred, she was not in metabolic decompensation. The temporal relation leads us to postulate that the neuroimaging findings were a result of metabolic abnormalities during her childhood and adolescent years or due to undiagnosed subclinical seizures during metabolic decompensation.

The predilection of the temporal lobes to injury from hyperammonemia is well-described. There is a specific pattern of brain injury in UCDs [3]. Acute hyperammonemia causes reversible changes involving the deep sulci of the insular and perirolandic regions. MRI studies have described hypomyelination, myelination delay, and cystic changes of the white matter and gliosis of the deep gray matter, which occurred months after acute neonatal hyperammonemia as a result of OTC deficiency [4]. Bilateral anterior temporal atrophy and high signal intensity at the temporal cortex have been observed in patients with late onset OTC deficiency, especially in the cingulate gyri and insular cortex [5]. Other MRI findings in OTC deficiency in patients with severe clinical manifestations such as somnolence, seizures, and hemiplegia included extensive infarct-like abnormalities, which were not present in our patient [6].

Besides routine MRI (T1 and T2 imaging), other neuroimaging modalities have been used to detect earlier measures of brain injury. Routine imaging may only detect damage at the macroscopic level when the patient is symptomatic and may lag behind clinical signs. Diffusion tensor imaging (DTI), which examines the disruption of white matter integrity, functional MRI (fMRI) which maps changes in brain hemodynamics that correspond to cognitive tasks, and proton magnetic resonance spectroscopy (^1^H MRS) which is able to detect the concentration of neurotoxic byproducts in various areas of the brain [7] have been used to characterize brain injury in patients with UCDs. In ^1^H MRS, there is an increase in the glutamine/glutamate signal intensity accompanied by myo-inositol depletion. Other details of these advanced neuroimaging findings are beyond the scope of this case report.

Ammonia is converted to glutamine in astrocytes and being osmotically active, causes cytotoxic edema. It has been postulated by Kurihara *et al.* [5] that the T2 abnormalities on MRI may reflect diminished cerebral perfusion and early ischemia. 

The neuroimaging findings in other causes of hyperammonemia differ from that due to UCDs. For example, in hepatic encephalopathy, classic MRI abnormalities include high signal intensity in the globus pallidum on T1-weighted images. The pattern of MRI abnormalities may thus also be able to guide the clinician in the workup for hyperammonemia [8].

With the knowledge of the specific neuroimaging findings of our patient, we are able to better determine the etiology and prognosis of focal epilepsy. She will probably need long-term anti-epileptic medications for seizure control.

## Conclusions

To the best of our knowledge, the finding of bilateral mesial temporal sclerosis adds further evidence of the susceptibility of temporal lobes to injury from hyperammonemia. UCD can lead to mesial temporal sclerosis which leads to increased susceptibility of patients to seizures regardless of their metabolic state. This case also highlights the importance of neuroimaging in the workup of seizures in patients with UCD without metabolic decompensation.

## References

[1] Machado MC, Pinheiro da Silva F. Hyperammonemia due to urea cycle disorders: a potentially fatal condition in the intensive care setting. J Intens Care. 2014;2:22. 10.1186/2052-0492-2-22 PMC4407289 25908985

[2] Wolf NI, Bast T, Surtees R. Epilepsy in inborn errors of metabolism. Epileptic Disord. 2005;7(2):67–81. 15929908

[3] Gropman AL. Patterns of brain injury in inborn errors of metabolism. Semin Pediatr Neurol. 2012;19(4):203–10. 10.1016/j.spen.2012.09.007 PMC3758694 23245553

[4] Gropman AL. Brain imaging in urea cycle disorders. Mol Genet Metab. 2010;100(Suppl 1):S20–30. 10.1016/j.ymgme.2010.01.017 PMC3258295 20207564

[5] Kurihara A, Takanashi J, Tomita M, Kobayashi K, Ogawa A, Kanazawa M, et al. Magnetic resonance imaging in late-onset ornithine transcarbamylase deficiency. Brain and Development. 2003;25:40–4. 10.1016/s0387-7604(02)00153-5 12536032

[6] Mamourian AC, du Plessis A. Urea cycle defect: a case with MR and CT findings resembling infarct. Pediatr Radiol. 1991;21:594–5. 10.1007/BF02012608 1815186

[7] Gropman AL. Expanding the diagnostic and research toolbox for inborn errors of metabolism: the role of magnetic resonance spectroscopy. Mol Genet Metab. 2005;86:2–9. 10.1016/j.ymgme.2005.07.009 16276565

[8] Rovira A, Alonso J, Cordoba J. MR imaging findings in hepatic encephalopathy. AJNR Am J Neuroradiol. 2008;29(9):1612–21. 10.3174/ajnr.A1139 PMC8118773 18583413

